# High-Performance TiO_2_ Nanotubes/Poly(aryl ether sulfone) Hybrid Self-Cleaning Anti-Fouling Ultrafiltration Membranes

**DOI:** 10.3390/polym11030555

**Published:** 2019-03-23

**Authors:** Zhi Geng, Xinyu Wang, Hongchuan Jiang, Leilei Zhang, Zhiting Chen, Yong Feng, Wenzhe Geng, Xia Yang, Mingxin Huo, Jing Sun

**Affiliations:** 1 College of Environment, Research Centre for Municipal Wastewater Treatment and Water Quality Protection, Northeast Normal University, Changchun 130117, China; gengz100@nenu.edu.cn (Z.G.); wangxy275@nenu.edu.cn (X.W.); jianghc331@nenu.edu.cn (H.J.); zhangll554@menu.edu.cn (L.Z.); fengy234@nenu.edu.cn (Y.F.); gengwz595@nenu.edu.cn (W.G.); huomx097@nenu.edu.cn (M.H.); 2 School of Chemistry and Environmental Engineering, Changchun University of Science and Technology, Changchun 130022, China; sunjing@cust.edu.cn; 3 College of Ecology and Resources Engineering, Wuyi University, Wuyishan 354300, China; wysczt@wuyiu.edu.cn

**Keywords:** poly(aryl ether sulfone), self-cleaning, ultrafiltration, photocatalytic, titanium dioxide nanotubes

## Abstract

A series of novel self-cleaning hybrid photocatalytic ultrafiltration (UF) membranes were fabricated to separate polyacrylamide, which is widely used as a commercial flocculant. To maximize the self-cleaning and anti-fouling properties of hybrid membranes, high surface area TiO_2_ nanotubes (TNTs) with excellent photocatalytic activity were homogeneously introduced into a poly(aryl ether sulfone) matrix by chemical bonds. The chemical structure, micromorphology, hydrophilicity, separation efficiency, fouling behavior, and self-cleaning property of the prepared hybrid membranes were well characterized and evaluated. For the optimal sample, the flux recovery ratio increased from ~40% to ~80% after simulated sunlight irradiation for 20 min, which was attributable to the homogeneous dispersion and efficient photocatalytic degradation ability of TNTs. Furthermore, the intelligent fabrication strategy enhanced the anti-aging ability of the hybrid membranes via the use of a fluorine-containing poly matrix. This work provided new insight into the fabrication of high-performance self-cleaning inorganic/organic hybrid membranes.

## 1. Introduction

Water scarcity and contamination due to the development of industrialization and population growth have gained extensive attention worldwide [1,2]. Membrane separation technology is an attractive technology for water treatment because of its environmentally friendly nature, non-phase-transition procedure, as well as ease of operation and scale-up, etc. [3,4,5,6,7,8]. As a type of membrane separation technology, an ultrafiltration (UF) membrane offers several advantages such as high efficiency of removal of macromolecular organic compounds ans relatively low operation pressure and energy consumption compared to nanofiltration (NF) and reverse osmosis (RO) membranes [9,10,11].

However, one major obstacle for the widespread application of ultrafiltration is membrane fouling which causes a dramatic decrease in the water flux and separation efficiency due to the adsorption and deposition effects of pollutants on the membrane surface or within membrane pores [12,13,14]. Therefore, various approaches have been attempted to reduce UF membrane fouling, like surface grafting with hydrophilic monomers [15], blending of amphiphilic polymer in the polymer matrix [16], polymer functionalization [17], and embedding inorganic nanomaterials into polymer matrices [18,19,20]. Among these methods, introducing inorganic photocatalytic nanomaterials into the polymer matrix to fabricate composite photocatalytic UF membranes has attracted enormous interests in recent years due to their unique properties, for example, anti-fouling, anti-microbial, self-cleaning, concurrent photocatalytic oxidation and separation, which could potentially overcome the drawbacks associated with conventional UF membranes. Wang et al. prepared Fe-doped TiO_2_/PSF (polysulfone) composite UF membranes by blending different amounts of Fe-TiO_2_ photocatalyst into polysulfone (PSF) membranes and investigated their bisphenol A (BPA) removal performances, mechanical properties, and self-cleaning abilities. The results displayed that the water flux, fouling prevention, and mechanical capacity of the composite UF membranes were enhanced compared to pure PSF membranes. In addition, the composite UF membranes exhibited a high BPA degradation rate under visible-light irradiation [21]. Song et al. manufactured LiCl-TiO_2_-doped PVDF (polyvinylidene fluoride) UF membranes by incorporating an inorganic pore-forming agent, LiCl, and TiO_2_ nanoparticles into the PVDF casting solution. The prepared composite UF membranes demonstrated a better retention rate for natural organic matter (NOM) and self-cleaning properties compared with neat PVDF membrane [22]. Fischer et al. incorporated TNTs into a polyethersulfone (PES) microfiltration membrane via anodization of TNT on the surface of PES membranes. They reported that the TNT-PES membranes showed high photocatalytic degradation activity of diclofenac and could fully degrade diclofenac to non-toxic products [23].

However, some design and fabrication issues still need to be investigated and resolved before photocatalytic UF membranes can be practically applied for water purification [24]. Firstly, more efforts are required to consider a new strategy for fabricating a high catalytic activity hybrid UF membrane by introducing photocatalysts with a high specific surface area and simultaneously ensuring their homogeneous dispersion in the polymer matrix. Secondly, avoiding the detachment of photocatalysts as much as possible during the water treatment process must be explored. In addition, the anti-aging ability should be enhanced as for inorganic-polymer composite photocatalytic UF membranes, the aging of the polymers may be accelerated by photocatalytic degradation, in that case, the separation efficiency of membranes will be affected significantly.

Titanium dioxide nanotubes (TNTs) are one of the most widely investigated photocatalytic nanomaterials due to their high specific surface area and catalytic activity, good chemical and thermal stability, and low toxicity [25,26]. Especially, TNTs can effectively degrade organic contaminants into less toxic or biodegradable organic compounds, even mineralize them into CO_2_, H_2_O, or other small molecule inorganics [27,28]. A PES membrane is widely used in membrane separation fields because of its outstanding mechanical property and thermal and chemical stabilities. However, commercial PES membranes are prone to fouling during the water treatment process due to their hydrophobic nature [29,30,31].

To sum up, in the present study, high specific surface area and catalytic activity photocatalyst TNTs were successfully prepared by, firstly, an alkaline hydrothermal method of commercial P25 precursor. Then, a PES-F-COOH polymer matrix which contained trifluoromethyl and carboxyl groups was synthesized through the copolymerization of 4,4′-(hexafluoroisopropylidene) diphenol (HFID), phenolphthalein (PPL), and 4,4′-difluorophenyl sulfone (DFPS). Introducing trifluoromethyl groups into the backbone of the polymer chain could endow PES-F-COOH with excellent photocatalytic aging resistance due to the high bond energy of C-F. The delicate molecular design makes PES-F-COOH a promising candidate for photocatalyst carriers [32,33,34]. Finally, a series of novel inorganic-polymer TNTs/PES-F-COOH hybrid photocatalytic UF membranes were fabricated by grafting TNTs onto the side chain of the PES-F-COOH matrix using a silane coupling agent. The strong covalent bonds between the inorganic filler and organic polymer matrix ensure the high-specific-surface area TNTs homogeneously disperse within the PES-F-COOH matrix and that the photocatalysts can exhibit remarkable photocatalytic activities in terms of degrading a series of organic pollutants. Additionally, the detachment of photocatalysts is significantly inhibited during the water treatment process. The chemical structure, micromorphology, and hydrophilicity of the prepared TNT/PES-F-COOH hybrid photocatalytic UF membranes were well characterized. Further, the separation efficiency, fouling behavior, and self-cleaning property of the above-mentioned membranes were investigated using a polyacrylamide (PAM) foulant solution by dead-end filtration experiments. Additionally, the anti-photocatalytic aging properties of the hybrid UF membranes were also evaluated.

## 2. Materials and Methods

### 2.1. Materials

Phenolphthalein (PPL) was provided by TCI Shanghai Development Co. Ltd., China. The reagents 4,4′-(hexafluoroisopropylidene) diphenol (HFID), 4,4′-difluorophenyl sulfone (DFPS), polyvinylpyrrolidone (PVP), titanium dioxide (P25 TiO_2_), and polyacrylamide (PAM, Mw: 2.0 × 10^6^–1.4 × 10^7^), were purchased from Aladdin Reagent Shanghai Co. Ltd. (Shanghai, China). *N*,*N*-dimethylformamide (DMF) and tetrahydrofuran (THF) were also purchased from Aladdin Reagent Shanghai Co. Ltd., and further purified according to the standard processes. Anhydrous potassium carbonate (K_2_CO_3_), toluene, and oxalyl chloride were supplied by Sinopharm Chemical Reagent (Shanghai, China). γ-Aminopropyltriethoxysilane (KH-550) was received from Nanjing Fine Chemical Co. Ltd. (Nanjing, China). Tetramethylene sulfone (TMS) was procured from Energy Chemical Shanghai Co. Ltd. (Shanghai, China) and further purified by vacuum distillation. All other chemicals were of reagent grade and obtained from commercial sources. Deionized (DI) water with a minimum resistance of 18 MΩ·cm was used throughout the experiments.

### 2.2. Synthesis of Titanium Dioxide Nanotubes (TNTs) Photocatalysts

TNTs were prepared by an alkaline hydrothermal method using a commercial P25 TiO_2_ precursor [26]. The synthesis process was as follows: P25 TiO_2_ (0.6 g) and NaOH solution (30 mL, 10 M) were stirred at room temperature for 1 h and then the resulting mixture was poured into a Teflon-lined autoclave at 150 °C for 12 h. After cooling to room temperature, the sample was separated by centrifugation. The obtained white powder was washed with HCl solution, DI water, and ethanol to neutrality and then dried at 80 °C for 12 h. Finally, the product was calcined at 400 °C for 2 h in a muffle furnace and subsequently ground to obtain TNTs.

### 2.3. Synthesis of PES-F-COOH Copolymer

**Scheme 1:**
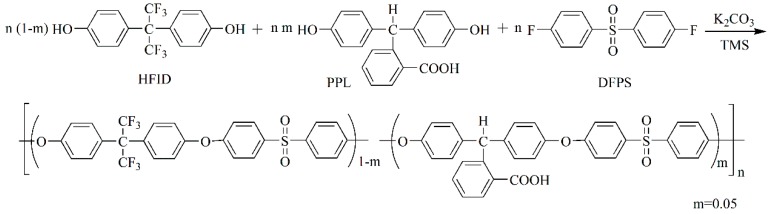
Synthesis of the polyethersulfone (PES)-F-COOH copolymer.

The anti-photocatalytic aging PES-F-COOH copolymer containing trifluoromethyl and carboxyl groups was synthesized via aromatic nucleophilic substitution copolymerization, as shown in Scheme 1 [14]. The entire experiment was conducted under nitrogen protection. HFID (12.78 g, 0.038 mol), PPL (0.64 g, 0.002 mol), DFPS (10.16 g, 0.040 mol), anhydrous K_2_CO_3_ (5.8 g, 0.042 mol), TMS (70 mL), and toluene (35 mL) were mixed in a three-necked flask with a mechanical stirrer, a nitrogen inlet with a thermometer, and a Dean–Stark trap with a condenser. The mixture was stirred at room temperature for about 10 min and then heated to 125 °C. After 2 h of reaction under reflux, the residual water and excess toluene were removed from the Dean–Stark trap. The reaction temperature was increased to 165–175 °C and maintained for 5 h for the copolymerization procedure. After naturally cooling to room temperature, the viscous solution was poured into DI water to obtain a flexible threadlike polymer. Then, the products were broken into powder by a high-speed blender, and the powder was washed five times with hot DI water and ethanol, respectively. After drying, the powder was redissolved in THF and acidified with HCl solution. Finally, the products were washed to neutrality with DI water and vacuum dried at 80 °C for 48 h to obtain PES-F-COOH copolymer. M_w_: 5.12 × 10^4^, M_n_: 2.64 × 10^4^, polydispersity index (PDI): 1.94, as determined by gel permeation chromatograms (GPC).

### 2.4. Synthesis of TNTs/PES-F-COOH Hybrid Materials

**Scheme 2:**
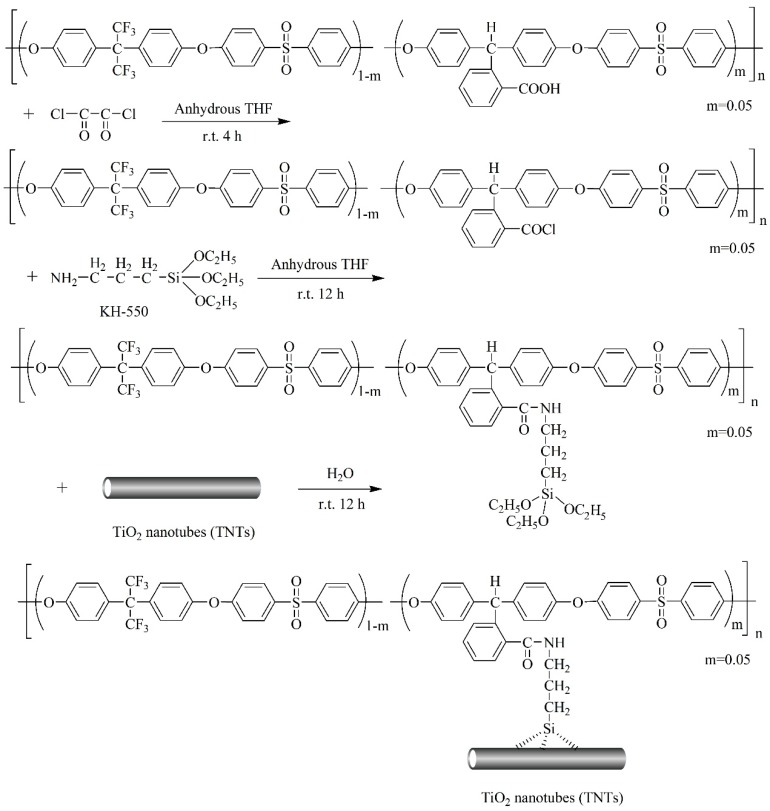
Synthesis of titanium dioxide nanotubes (TNTs)/PES-F-COOH hybrid materials.

The preparation method of TNTs/PES-F-COOH hybrid materials was shown in Scheme 2. Previously prepared PES-F-COOH copolymer (2 g) was dissolved in 20 mL anhydrous tetrahydrofuran (THF) and, after stirring for 1 h at room temperature, 0.06 mL oxalyl chloride was added to the polymer solution. The mixture was stirred for 4 h at room temperature to ensure the complete conversion of carboxyl groups to acyl chloride groups. Subsequently, the excess oxalyl chloride and THF solvent were removed using vacuum distillation. The obtained product was redissolved in 20 mL anhydrous tetrahydrofuran, and 0.06 mL KH-550 was injected into the reactor. The mixture was stirred for 12 h at room temperature. Then, TNTs (1 wt%, 3 wt%, and 5 wt% relative to the weight of PES-F-COOH) and 0.012 mL DI water were added. After another 12 h stirring at room temperature, the white, flexible, threadlike raw product was obtained by pouring the resulting suspension into DI water. The raw product was then pulverized into a powder using a high-speed blender, and the powder was washed several times with DI water and ethanol, respectively. Finally, 1%TNTs/PES-F-COOH, 3%TNTs/PES-F-COOH, and 5%TNTs/PES-F-COOH hybrid materials were obtained after vacuum drying at 80 °C for about 48 h.

### 2.5. Preparation of TNTs/PES-F-COOH Ultrafiltration Membranes

A series of TNTs/PES-F-COOH ultrafiltration membranes with different contents of TNTs were fabricated via a non-solvent-induced phase inversion method [35]. Typically, 2 g 5%TNTs/PES-F-COOH hybrid material and 0.2 g PVP were dissolved in 10 mL DMF to form a homogeneous casting solution. After degassing, the solution was poured onto a clean glass plate with polyester nonwoven fabric. The glass plate was evaporated in air for 60 s and then immersed into a water bath at room temperature to obtain 5%TNTs/PES-F-COOH ultrafiltration membrane.

### 2.6. Characterization

The molecular weight of the PES-F-COOH copolymer was investigated by gel permeation chromatograms (GPC, Waters 410, Milford, MA, USA) using polystyrene as a standard and tetrahydrofuran (THF) as an eluent at a flow rate of 1 mL min^−1^. The morphologies of TNTs were observed by a JEM-2100F high-resolution transmission electron microscope (TEM, JEOL, Tokyo, Japan) at an accelerating voltage of 200 kV. The chemical structures of hybrid materials were characterized by FT-IR spectra using a Fourier transform infrared spectrometer (Nicolet Impact 410, Madison, Wi, USA). A scanning electron microscope (SEM, FEI XL30, Eindhoven, the Netherlands) equipped with an energy dispersive spectrometer (EDS) detector was used to observe the morphologies of the ultrafiltration membranes. The contact angles (CA) of ultrafiltration membranes were measured by a goniometer (DSA 25, KRÜSS, Germany) equipped with a computer-controlled video camera using the sessile drop method. The dynamic contact angles were recorded for 30 s, and the water droplet images were captured by a video camera at the speed of 15 photos per second at room temperature. Each contact angle was obtained 2 s after a 3-μL DI water droplet equilibrated on the membrane surface. The mean value of the contact angle was calculated using contact angle data from six random locations of three membrane samples. A ultraviolet (UV)/visible (Vis) spectrophotometer (Puxi T6, New Century, Beijing, China) was used to measure the concentration of PAM solution at a wavelength of 470 nm with acetic acid (CH_3_COOH) and sodium hypochlorite (NaClO) as chromogenic agents. The photocatalytic self-cleaning and anti-photocatalytic aging properties of ultrafiltration membranes were evaluated under simulated sunlight irradiation (320 nm < λ < 680 nm) using a 300 W Xe lamp with an IR cut filter to block IR irradiation between 680 nm and 1100 nm. Experiments were performed in a cylindrical quartz photocatalytic reactor equipped with a temperature-controlled water circulation bath.

### 2.7. Ultrafiltration Tests

The separation efficiency, fouling behavior, self-cleaning and anti-photocatalytic aging properties of the TNTs/PES-F-COOH hybrid ultrafiltration membranes were evaluated by (1) measuring the pure water flux (*J_w_*), water flux in the presence of PAM foulant (*J_p_*), retention rate (*R*) and (2) calculating the total fouling ratio (*R_t_*), reversible fouling ratio (*R_r_*), irreversible fouling ratio (*R_ir_*), and flux recovery ratio (*F_RR_*). These parameters were evaluated by ultrafiltration experiments using a PAM foulant solution (*C_f_* = 1000 mg L^−1^) in a dead-end ultrafiltration setup (MSC-50, Shanghai Mosu Science Instruments Co., Ltd.) with an effective membrane area (*A*) of 9.6 cm^2^. The procedures of ultrafiltration experiments were as follows: (1) First, the membranes were pre-compacted using DI water under a pressure of 0.15 MPa for 20 min to get a stable flux of DI water; (2) the initial pure water flux (*J*_*w*,0_) was calculated by recording the volume of permeate DI water (*V*_*w*,0_) per minute under a pressure of 0.15 MPa for 10 min; (3) water flux in the presence of PAM (*J*_*p*,1_) was calculated by measuring the volume of permeate liquid (*V*_*p*,1_) every three minutes under a pressure of 0.15 MPa for 30 min; (4) the retention rate (*R*_1_) was calculated by detecting the concentration of PAM in the permeate solution (*C*_*p*,1_); (5) after ultrasonic cleaning with DI water for 20 min, the pure water flux (*J*_*w*,1_) was reevaluated by the same method used for *J*_*w*,0_; (6) the pure water flux (*J*_*w*,2_) and PAM retention rate (*R*_2_) were reevaluated by the same method as *J*_*w*,0_ and *R*_1_, respectively, after the membranes were exposed under simulated sunlight for 20 min without filtration.

The pure water flux (*J_w_*) and water flux in the presence of PAM (*J_p_*) was calculated from:

(1) $$J=\frac{V}{At}$$

where *V* is the permeate volume, *A* is the effective membrane area, and *t* is the filtration time.

The retention rate (*R*) was calculated from:

(2) $$R(\%)=\left(1-\frac{C_{p}}{C_{f}}\right)\times 100$$

where *C_p_* and *C_f_* are the PAM concentrations in the permeate and feed solution, respectively.

The total fouling ratio (*R_t_*), reversible fouling ratio (*R_r_*), and irreversible fouling ratio (*R_ir_*) before and after simulated sunlight irradiation were calculated by the following equations, respectively:

(3) $$R_{t}(\%)=\left(\frac{J_{w,0}-J_{p,1}}{J_{w,0}}\right)\times 100$$

(4) $$R_{r}(\%)=\left(\frac{J_{w,n}-J_{p,1}}{J_{w,0}}\right)\times 100$$

(5) $$R_{ir}(\%)=\left(\frac{J_{w,0}-J_{w,n}}{J_{w,0}}\right)\times 100$$

where *J*_*w*,0_ is the initial pure water flux, *J*_*p*,1_ is the water flux in the presence of PAM foulant in feed solution, *J_w,n_* is the pure water flux after ultrasonic cleaning with DI water for 20 min (*J*_*w*,1_) or pure water flux after simulated sunlight irradiation for 20 min (*J*_*w*,2_).

The flux recovery ratio (*F_RR_*) was calculated from:

(6) $$F_{RR}(\%)=\frac{J_{w}}{J_{w,0}}\times 100$$

where *J_w_* is the pure water flux after ultrasonic cleaning of the membranes with DI water for 20 min (*J*_*w*,1_) or exposing under simulated sunlight for 20 min (*J*_*w*,2_), *J*_*w*,0_ is the initial pure water flux.

### 2.8. Mean Pore Size and Prosity Tests

The mean pore size of membranes was measured by a modified bubble point method and calculated using the following equation:

(7) $$r=\frac{2\sigma \cos\theta }{P}$$

where *σ* is the interfacial tension of water/water-saturated isobutanol (*σ* = 1.7 dyne/cm), *θ* is the membrane water contact angle, and *P* is the applied pressure.

The porosities (*Po*) of the membranes were measured according to the membrane weight changes and calculated using the following equation:

(8) $$Po(\%)=\left(\frac{M_{w}-M_{d}}{S\cdot d\cdot \rho }\right)\times 100$$

where *M_w_* is the weight of a membrane filled with water, *M_d_* is the weight of a dry membrane, *S* is the area of a membrane, *d* is the average thickness of a membrane, and *ρ* is the density of water.

## 3. Results and Discussion

### 3.1. Morphology of TNTs Catalyst

**Figure 1:**
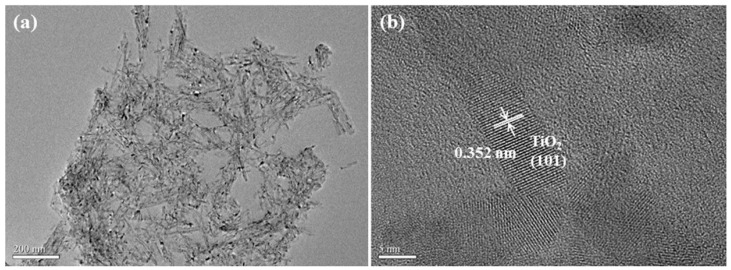
Transmission electron microscope (TEM) images of TNTs. (**a**) low-magnification; (**b**) high-magnification.

TNTs were successfully synthesized by the alkaline hydrothermal method, as shown in Figure 1. As can be seen from Figure 1a, a larger number of TNTs had a cylindrical shape with a hollow cavity and were about 200 to 300 nm in length with a diameter of around 10 nm. A high-magnification TEM image further revealed the highly crystalline nature of anatase TiO_2_ TNTs which were formed, and this was proved by the lattice spacing of 0.352 nm, which corresponded to the (101) plane of TiO_2_, Figure 1b. The possible formation mechanism of TNTs by P25 precursor contained two processes, exfoliating and rolling, as reported by other researchers [36,37,38,39,40].

### 3.2. Chemical Structures of TNTs/PES-F-COOH Hybrid Materials

**Figure 2:**
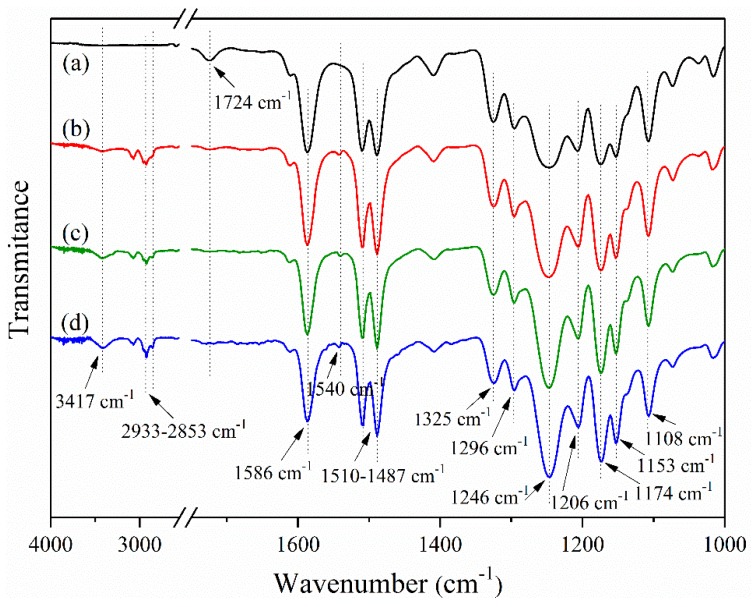
FT-IR spectra of (**a**) PES-F-COOH copolymer matrix, (**b**) 1%TNTs/PES-F-COOH hybrid material, (**c**) 3%TNTs/PES-F-COOH hybrid material, (**d**) 5%TNTs/PES-F-COOH hybrid material.

The FT-IR spectra of the PES-F-COOH copolymer matrix and TNTs/PES-F-COOH hybrid materials are shown in Figure 2, revealing the following: 1586 cm^−1^ and 1510–1487 cm^−1^ (C=C bond of benzene ring), 1325 cm^−1^, 1296 cm^−1^, and 1153 cm^−1^ (O=S=O bond of aromatic sulfone in the main chain), 1246 cm^−1^ (aromatic ether linkage Ar–O–Ar in the main chain), 1206 cm^−1^ and 1174 cm^−1^ (–CF_3_ groups in the side chain), 1108 cm^−1^ (Ar–S–Ar groups in the main chain). Further, by comparison with the FT-IR spectrum of the PES-F-COOH copolymer, shown in Figure 2a, it was found that the characteristic absorbance peak at 1724 cm^−1^ ascribed to the C=O bond of aromatic carboxyl groups (–COOH) disappeared; meanwhile, a peak from 2933 to 2853 cm^−1^ attributed to the methylene C–H bond of KH-550 and a peak at 1540 cm^−1^ associated with the N–H bond of acid amide (–CO–NH–) appeared in the FT-IR spectra of the TNTs/PES-F-COOH hybrid materials, see Figure 2b–d. This means that the –NH_2_ group of KH-550 reacted with the –COOH group of the PES-F-COOH copolymer to form –CO–NH– groups, and KH-550 was grafted onto the side chain of the copolymer. In addition, a new peak at 3417 cm^−1^ attributable to –OH group of TNTs clusters was also observed, see Figure 2b–d. This means that the TNTs clusters exist in hybrid materials. Hydroxyl (–OH) groups on the TNTs surface could easily react with Si(OH)_3_ generated from the hydrolysis of KH-550 to form Si–O–TNTs via a classic condensation reaction [14,41]. Therefore, the TNTs were grafted onto the side chain of the polymer matrix through the silane coupling agent, KH-550, as a bridge. To sum, the TNTs/PES-F-COOH hybrid materials were successfully synthesized.

### 3.3. Morphology of UF Membranes

**Figure 3:**
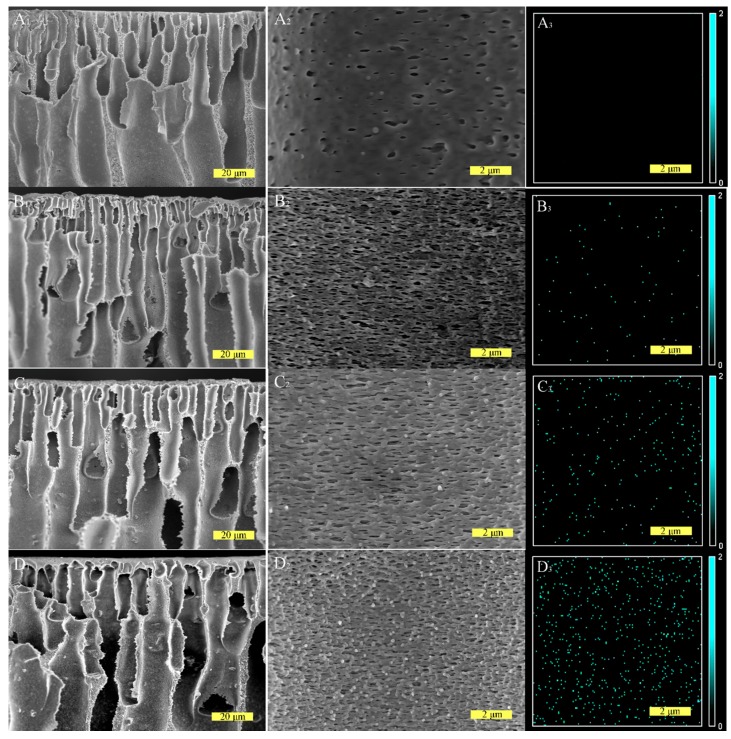
Cross-section SEM and Ti-mapping EDS images of the ultrafiltration membranes: (**A_1,2,3_**): pristine PES-F-COOH; (**B_1,2,3_**): 1%TNTs/PES-F-COOH; (**C_1,2,3_**): 3%TNTs/PES-F-COOH; and (**D_1,2,3_**): 5%TNTs/PES-F-COOH.

To investigate the effects of TNTs on the membrane microstructure, the cross-section SEM images of PES-F-COOH and TNTs/PES-F-COOH ultrafiltration membranes are compared in Figure 3. From the low-magnification SEM images, see Figure 3A_1_–D_1_, ×1000, it could be found that all the membranes showed an asymmetric structure consisting of a thin, compact skin layer and a porous supporting layer with fully developed finger-like macrovoids across the thickness [42,43]. We also observed that the void size of finger-like macrovoids grew slightly with the increasing content of TNTs, see Figure 3B_1_–D_1_. This phenomenon is likely due to the fact that the incorporation of hydrophilic TNTs increased the solvent/non-solvent exchange rate during the phase inversion process of UF membrane preparation, thereby facilitating the formation of larger sized pores.

The high-magnification SEM images (×10,000) clearly displayed that the pristine PES-F-COOH membrane had cellular structured pores, see Figure 3A_2_. However, the TNTs/PES-F-COOH membranes exhibited lace-like structures with higher porosity, as shown in Figure 3B_2_–D_2_. The formation of a lace-like structure mainly contributed to the transformation of the rheological properties from Newtonian fluid to non-Newtonian fluid of the casting solution due to the strong interaction between hybrid polymer molecules which contain TNTs with high specific surface area and surface energy. On the other hand, for TNTs which provided abundant reactive sites for the silane coupling agent, multiple polymer chains were cross-linked to the TNTs via a side-chain grafting reaction [44,45]. As we expected, no aggregated TNTs clusters were detected in the SEM images of TNTs/PES-F-COOH hybrid membranes, see Figure 3B_2_–D_2_.

Further, the dispersion and content of TNTs within the UF membranes were indicated by EDS titanium (Ti) mapping analysis, see Figure 3A_3_–D_3_, ×10,000 magnification. No signal was observed in the Ti-mapping image of the pure PES-F-COOH membrane, see Figure 3A_3_. While the aqua dots, which represented the titanium element in TNTs, were clearly observed in the Ti-mapping images of TNTs/PES-F-COOH hybrid UF membranes, see Figure 3B_3_–D_3_. The results illustrated that the TNTs were successfully incorporated and well-dispersed in the hybrid UF membranes. Furthermore, the density of the aqua dots correlated well with the TNTs content in the hybrid UF membranes. Based on the SEM and Ti-mapping EDS images results, we found the compatibility of inorganic TNTs and the organic polymer matrix was significantly improved, likely due to the covalent binding of TNTs on the side chains of the polymer matrix achieved by the silane coupling agent KH-550.

### 3.4. Membrane Hydrophilicity

**Figure 4:**
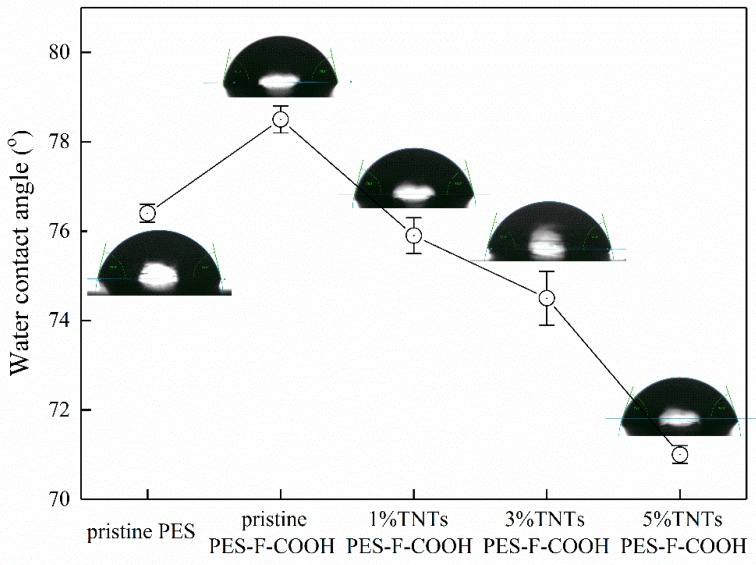
Water contact angle variations of the pristine PES, pristine PES-F-COOH, 1%TNTs/PES-F-COOH, 3%TNTs/PES-F-COOH, and 5%TNTs/PES-F-COOH ultrafiltration membranes.

The hydrophilicity and wettability of UF membrane compact skin layer materials play an important role in controlling membrane water transport and alleviating membrane fouling in the process of separation. To investigate the effects of introducing trifluoromethyl groups and TNTs addition on membrane surface hydrophilicity and wettability, water contact angles of the pristine PES, pristine PES-F-COOH, and TNTs/PES-F-COOH ultrafiltration membranes were measured; the results are summarized in Figure 4. Introducing trifluoromethyl groups into the polymer chain results in hydrophobicity, so the pristine PES-F-COOH membrane exhibited a higher water contact angle (average of 78.5°) compared with that of the pristine PES membrane (average of 76.4°). As for TNTs/PES-F-COOH hybrid membranes, introducing trifluoromethyl groups lead to hydrophobicity on the one hand, and on the other hand, the addition of TNTs with plenty of hydroxyl groups resulted in remarkable hydrophilicity. As the increasing hydrophilicity plays a dominant role in hybrid membranes, the TNTs/PES-F-COOH membranes exhibited lower water contact angles compared to the pristine PES membrane. Moreover, the water contact angles of TNTs/PES-F-COOH membranes gradually decreased (average of 75.9–71.0°) with a TNTs content from 1 wt% to 5 wt%. A lower water contact angle also represented a greater tendency for water to wet the membrane. Therefore, the results confirmed that the hydrophilicity and wettability of hybrid ultrafiltration membranes could be improved by the incorporation of TNTs into the polymer chain during hybrid material fabrication.

### 3.5. Membrane Separation Performance

**Figure 5:**
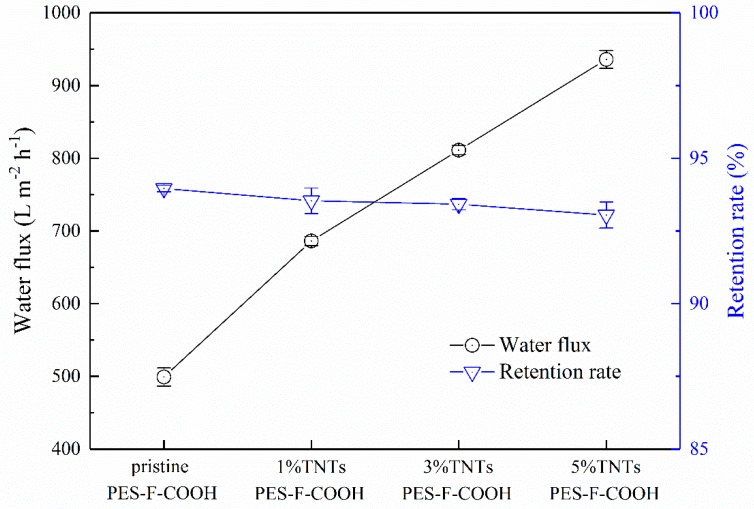
Water flux and polyacrylamide (PAM) retention rate of the pristine PES-F-COOH, 1%TNTs/PES-F-COOH, 3%TNTs/PES-F-COOH, and 5%TNTs/PES-F-COOH ultrafiltration membranes.

To evaluate the effects of TNTs addition on the membrane separation performance, the pure water flux and retention rate were measured by dead-end ultrafiltration experiments using DI water and 1000 mg L^−1^ PAM solution as a feed, respectively, at an applied pressure of 0.15 MPa. As presented in Figure 5, a substantial increase in the pure water flux and a slight concomitant decrease in the PAM rejection rate of UF membranes were observed with the increasing TNTs content. The pure water flux significantly increased from 499 L m^−2^ h^−1^ to 936 L m^−2^ h^−1^ with an increasing TNTs content from 0 wt% to 5 wt%. This phenomenon may be due to the enhanced surface hydrophilicity and wettability, see Figure 4. Moreover, the increased mean pore size and porosity, see Table 1, also improved the pure water flux. On the other hand, the PAM retention rate of the UF membranes was reduced from 94% to 93% with an increasing TNTs content from 0 wt% to 5 wt%; however, the lowest retention rate for the membrane with 5 wt% TNTs content (i.e., the highest TNTs loading amount) is still acceptable. The small decrease in PAM rejection is likely attributable to the slight increase in mean pore size, see Table 1, of the TNTs/PES-FCOOH hybrid membranes. The observed relatively high PAM retention rates of hybrid membranes can be put down to TNTs tightly bonding to the polymer matrix through covalent bonds and the formation of homogeneous hybrid materials. Based on the above, the fabricated TNTs/PES-F-COOH hybrid membranes have an improved separation performance.

**Figure 6:**
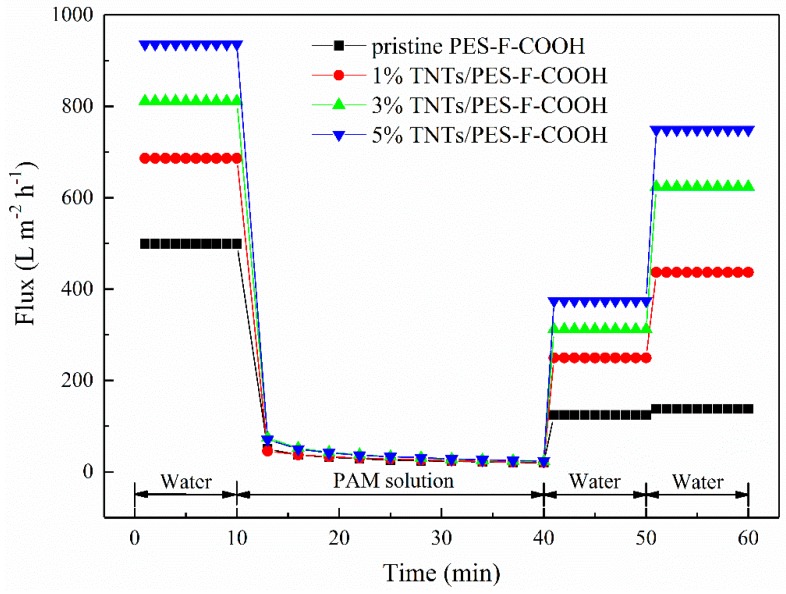
Variation in water flux with time for the pristine PES-F-COOH and TNTs/PES-F-COOH ultrafiltration membranes obtained following the procedures of Section 2.7 ultrafiltration tests.

To further investigate the membrane fouling and self-cleaning behaviors, the performances of the pristine PES-F-COOH and TNTs/PES-F-COOH membranes were measured and calculated (using Equation (1)) in terms of pure water flux, water flux in the presence of PAM foulant in feed solution, pure water flux after ultrasonic cleaning with DI water, and pure water flux after the membranes were exposed under simulated sunlight; the results are summarized in Figure 6. Compared to the initial high pure water flux, the PAM solution fluxes for both PES-F-COOH and TNTs/PES-F-COOH membranes sharply reduced to ~30 L m^−2^ h^−1^. That may be due to the formation of the PAM gel layer on the membrane surface at the beginning of dead-end filtration experiments. Such a fouling layer caused a great drop in flux upon the commencement of the fouling experiments. Although all the membranes demonstrated similar water flux in the presence of PAM foulant, the pure water fluxes after hydraulic cleaning and after exposure to simulated sunlight greatly increased with the increasing TNTs content. This result indicated excellent fouling reversibility and self-cleaning ability for the TNTs/PES-F-COOH hybrid membranes. The specific data and possible reasons will be discussed in detail in the following subsections.

### 3.6. Membrane Anti-Fouling Property

**Figure 7:**
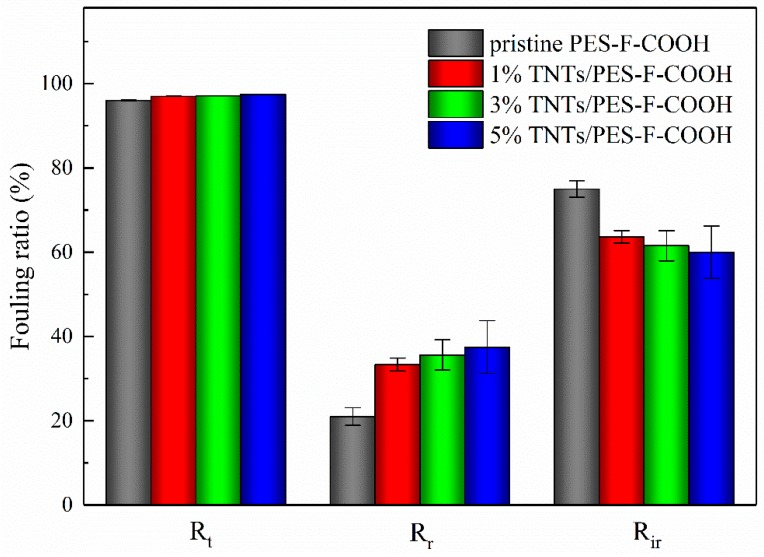
Total fouling ratio (*R_t_*), reversible fouling ratio (*R_r_*), and irreversible fouling ratio (*R_ir_*) before simulated sunlight irradiation of the pristine PES-F-COOH and TNTs/PES-F-COOH ultrafiltration membranes during ultrafiltration tests when 1000 mg L^−1^ PAM foulant in feed solution.

**Figure 8:**
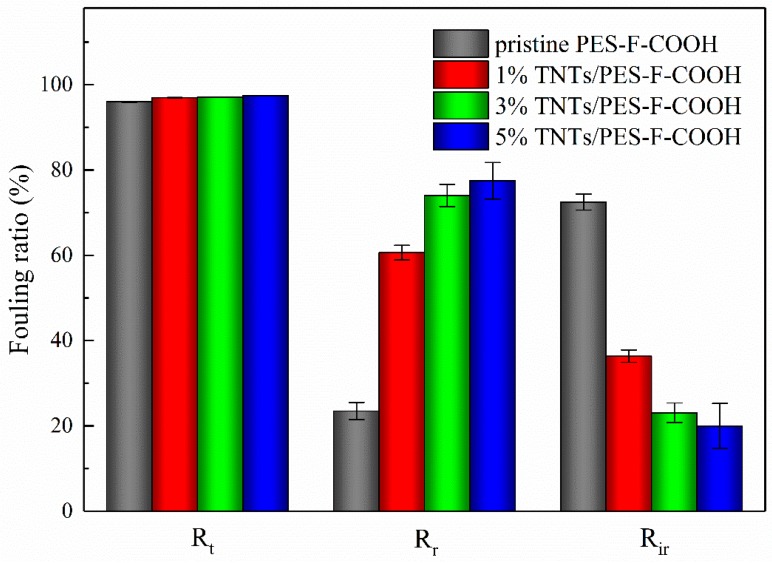
Total fouling ratio (*R_t_*), reversible fouling ratio (*R_r_*), and irreversible fouling ratio (*R_ir_*) after simulated sunlight irradiation of the pristine PES-F-COOH and TNTs/PES-F-COOH ultrafiltration membranes during ultrafiltration tests when 1000 mg L^−1^ PAM foulant in feed solution.

It is well known that ultrafiltration membrane fouling is divided into reversible and irreversible fouling. As for reversible fouling caused by the absorbed foulants, this was easily removed by hydraulic cleaning. In contrast, it is a big challenge to remove the foulants of irreversible fouling, which are strongly absorbed on the membrane surface or entrapped within the membrane pores. For an ultrafiltration membrane, the irreversible fouling usually plays an important role in a severe deterioration of membrane performance [12,46,47]. In order to investigate the anti-fouling properties of the PES-F-COOH and TNTs/PES-F-COOH ultrafiltration membranes, the total fouling ratios (*R_t_*), reversible fouling ratios (*R_r_*), and irreversible fouling ratios (*R_ir_*) before and after simulated sunlight irradiation of the prepared membranes were calculated using Equations (3)–(5), respectively, based on the flux data presented in Figure 6, and the variation tendencies of the fouling ratio before and after simulated sunlight irradiation are shown in Figure 7 and Figure 8, respectively. There were negligible changes for the total fouling ratios of tested membranes (all above 96%), whereas the reversible and irreversible fouling ratios had significant variations. The pristine PES-F-COOH sample displayed the lowest reversible fouling ratio (~21% and ~23%) and the highest irreversible fouling ratio (~75% and ~73%) before and after simulated sunlight irradiation. For the TNTs/PES-F-COOH hybrid UF membranes, the reversible fouling ratio gradually increased from ~33% to ~37% (before simulated sunlight irradiation) and form ~61% to ~77% (after simulated sunlight irradiation), and the irreversible fouling ratio decreased from ~64% to ~60% (before simulated sunlight irradiation) and form ~36% to ~20% (after simulated sunlight irradiation) with the increasing TNTs content from 1% to 5%, which indicated that the incorporation of TNTs could improve the anti-fouling properties and endow self-cleaning properties. Incorporation of TNTs with hydroxyl groups by a covalent bond and the formation of homogeneous hybrid materials have proved highly effective in enhancing the hydrophilicity of the hybrid membranes, and are, therefore, conducive to the formation of a hydration shell between membranes and foulants during separation. Therefore, the PAM deposited on the hybrid membranes was more easily removed by hydraulic cleaning. As a result, the proportion of reversible pollution to total pollution for hybrid membranes increased, and the anti-fouling performance of TNTs/PES-F-COOH hybrid membranes improved before simulated sunlight irradiation. In addition, the reversible fouling ratio dramatically increased and the irreversible fouling ratio sharply decreased after simulated sunlight irradiation, which due to the self-cleaning properties of hybrid membranes and the details will be discussed in Section 3.7.

### 3.7. Membrane Self-Cleaning and Anti-Photocatalytic Aging Properties

**Figure 9:**
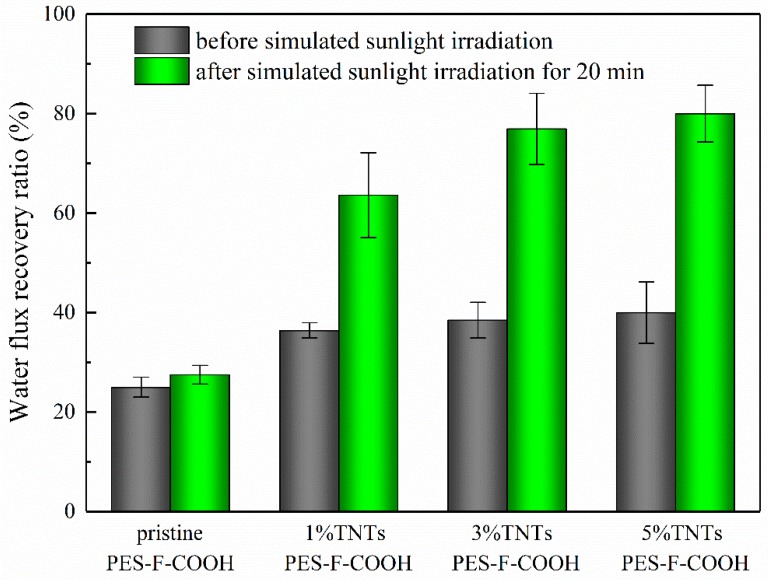
Water flux recovery ratios (F_RR_) for the pristine PES-F-COOH and TNTs/PES-F-COOH ultrafiltration membranes before and after simulated sunlight irradiation.

The self-cleaning performance of the TNTs/PES-F-COOH hybrid ultrafiltration membranes was evaluated by calculating the water flux recovery ratios (F_RR_, calculated from Equation (6)) before and after 20 min simulated sunlight irradiation. As shown in Figure 9, the ignored variation in F_RR_ was perceived for the pristine PES-F-COOH membrane, while a series of TNTs/PES-F-COOH hybrid membranes showed a drastic increase of F_RR_ after simulated sunlight irradiation. Especially for the 5%TNTs/PES-F-COOH hybrid membrane, the F_RR_ values before and after irradiation were ~40.0% and ~80.0%, respectively. It is evident that the increment of F_RR_ before and after irradiation increased significantly with the increasing TNTs content, and the highest increment of F_RR_ (~40%) was observed for the 5%TNTs/PES-F-COOH hybrid membrane. The self-cleaning performance of the reactive ultrafiltration membrane is very important for the water treatment efficiency and energy saving in the process of membrane separation, the fabricated TNTs/PES-F-COOH hybrid membranes exhibited superior self-cleaning properties compared to the similar state-of-the-art materials discussed in the previous literature [14]. The efficient self-cleaning capacity of the hybrid membranes was attributed to the hydroxyl radicals generated by the TNTs under irradiation, which degraded parts of the organic contaminants that were strongly attached to the membrane surface and trapped within the membrane pores [48,49]. Therefore, the self-cleaning mode of the hybrid membrane by photocatalytic degradation of organic contaminant is expected to be effective with regard to reducing irreversible PAM fouling. As researchers’ report, PAM degraded to acrylamide first, then part of acrylamide underwent demethylation and formed acetamide, and part of acrylamide lost a methyl and amino group to form acetic acid [50,51]. In addition, the homogeneous dispersion of the high specific surface area TNTs within the polymer matrix achieved by covalent bonding maintained the high photocatalytic activity of TNTs, thus endowing the hybrid membranes with excellent self-cleaning properties under sunlight irradiation.

**Figure 10:**
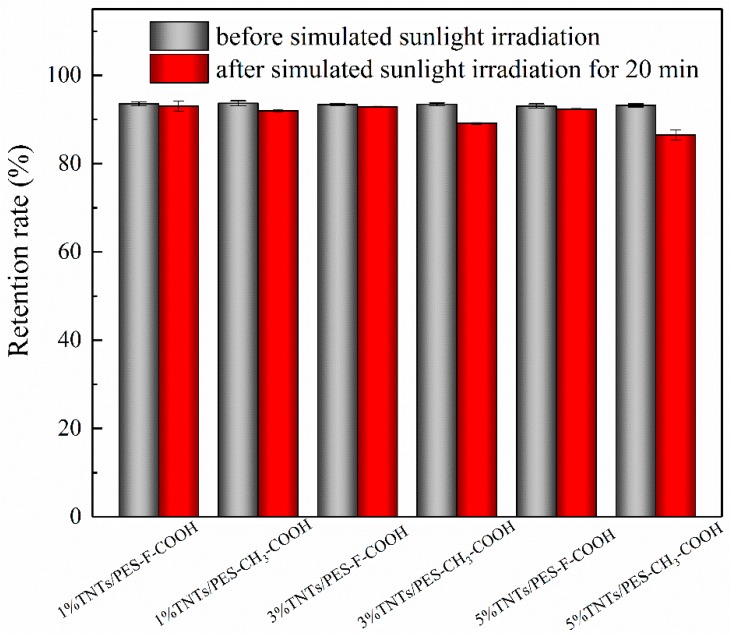
Retention rates for the fluorinated TNTs/PES-F-COOH and non-fluorinated TNTs/PES-CH_3_-COOH ultrafiltration membranes before and after simulated sunlight irradiation during ultrafiltration tests when 1000 mg L^−1^ PAM foulant in feed solution.

In order to demonstrate the anti-photocatalytic aging performance of the TNTs/PES-F-COOH ultrafiltration membranes and clarify the difference in the anti-photocatalytic aging ability between the fluorinated and non-fluorinated hybrid membranes. First, we chose bisphenol A (BPA) instead of HFID to synthesize the PES-CH_3_-COOH copolymer containing methyl and carboxyl groups according to the same procedure as that detailed in Section 2.3. After that, a series of non-fluorinated TNTs/PES-CH_3_-COOH hybrid ultrafiltration membranes were synthesized and prepared using the same procedure as that described in Section 2.4 and Section 2.5. Then, the PAM retention rates (R, calculated from Equation (2)) of fluorinated TNTs/PES-F-COOH and non-fluorinated TNTs/PES-CH_3_-COOH membranes before and after 20 min simulated sunlight irradiation were evaluated and compared; the results are summarized in Figure 10. As for fluorinated TNTs/PES-F-COOH hybrid membranes, comparable retention rates were observed before and after irradiation as was expected, and the retention rates after 20 min irradiation were above 92.3%. As for non-fluorinated TNTs/PES-CH_3_-COOH hybrid membranes, the retention rates were almost the same as those of TNTs/PES-F-COOH membranes before 20 min irradiation. However, it is clear that the retention rates obviously decreased with the increasing TNTs loading ratio after 20 min irradiation, and the retention rates after irradiation reduced to ~86.5% for the non-fluorinated 5%TNTs/PES-CH_3_-COOH sample. The above-mentioned results indicated that the fluorinated TNTs/PES-F-COOH hybrid membranes effectively resisted the deterioration in the self-cleaning photocatalytic oxidation process and displayed excellent anti-photocatalytic aging properties. Fluoropolymers were widely used as catalyst carriers and exhibited high photocatalytic aging resistance due to their C–F bonds with strong bonding energy [32,34,52]. Therefore, introducing a large amount of C–F bonds by the molecular design of fluoropolymer matrix in hybrid membranes inevitably enhanced the anti-photocatalytic aging ability.

## 4. Conclusions

An efficient strategy to fabricate high-performance self-cleaning hybrid ultrafiltration membranes was developed by the incorporation of TNTs with excellent photocatalytic activity into a fluorine-containing poly(aryl ether sulfone) matrix via covalent bonding. The strong interaction between the filler and polymer matrix maintains the homogeneous dispersion of high specific surface area TNTs, thus the self-cleaning effects of hybrid membranes were maximized. Meanwhile, the potential threat of TNTs nanomaterials being released into the environment was overcome. Furthermore, the prepared TNTs/PES-F-COOH hybrid ultrafiltration membranes also displayed remarkable separation, anti-fouling, and anti-photocatalytic aging ability. This method could be extended to other hybrid polymer systems containing high specific surface area photocatalysts, and these kinds of hybrid materials may have broad application prospects in the process of water treatment.

## Figures and Tables

**Table 1:** The mean pore size and porosity of the pristine PES-F-COOH and TNTs/PES-F-COOH hybrid ultrafiltration membranes.

Samples	Mean Pore Size (nm)	Porosity (%)
pristine PES-F-COOH	24.89	68.2
1%TNTs/PES-F-COOH	25.54	73.1
3%TNTs/PES-F-COOH	26.15	76.9
5%TNTs/PES-F-COOH	26.80	80.3
